# Prevalence of dyslipidaemia in statin-treated patients in South Africa: results of the DYSlipidaemia International Study (DYSIS)

**DOI:** 10.5830/CVJA-2013-071

**Published:** 2013-10

**Authors:** Frederick J Raal, Dirk J Blom, Shanil Naidoo, Peter Bramlage, Philippe Brudi

**Affiliations:** Carbohydrate and Lipid Metabolism Research Unit, Department of Medicine, University of the Witwatersrand, Johannesburg, South Africa; Division of Lipidology, Department of Medicine, University of Cape Town, Cape Town, South Africa; MSD South Africa, Midrand, South Africa; Institut für Pharmakologie und präventive Medizin, Mahlow, Germany; Merck, Sharp & Dohme Corp, Whitehouse Station, NJ, USA

**Keywords:** cardiovascular disease (CVD), dyslipidaemia, lipid abnormalities, statins, low-density lipoprotein cholesterol (LDL-C)

## Abstract

**Introduction and objectives:**

Cardiovascular disease (CVD) is the leading cause of mortality worldwide and increased levels of low-density lipoprotein cholesterol (LDL-C) are an important modifiable risk factor. Statins lower LDL-C levels and have been shown to reduce CVD risk. Despite the widespread availability of statins, many patients do not reach the lipid targets recommended by guidelines. We evaluated lipid goal attainment in statin-treated patients in South Africa and analysed variables contributing to poor goal attainment as part of the DYSlipidaemia International Study (DYSIS).

**Methods:**

This cross-sectional, observational study enrolled 1 029 consecutive South African patients consulting officebased physicians. Patients were at least 45 years old, had to be treated with a stable dose of statins for at least three months and had been fasting for 12 hours. We evaluated lipid goal attainment and examined variables associated with residual dyslipidaemia [abnormal levels of LDL-C, highdensity lipoprotein cholesterol (HDL-C) and/or triglycerides (TG)].

**Results:**

We found that 50.3% of the patients overall did not achieve target LDL-C levels and 73.5% of patients were at very high cardiovascular risk. In addition, 33.7% had low levels of HDL-C, while 45.3% had elevated TG levels despite statin therapy. Asian and mixed-ancestry patients but not black (vs Caucasian ethnicity), as well as obese individuals in South Africa were more likely to still have dyslipidaemia involving all three lipid fractions.

**Conclusions:**

We observed that many patients in South Africa experienced persistent dyslipidaemia despite statin treatment, supporting the concept that there is a need for more intensive statin therapy or the development of novel treatment strategies. Measures aimed at combating obesity and other lifestyle-related risk factors are also vital for effectively controlling dyslipidaemia and reducing the burden of CVD.

## Abstract

Cardiovascular disease (CVD) is the leading cause of mortality worldwide. In 2008, World Health Organisation (WHO) estimates suggested that 30% (17.3 million) of all deaths worldwide could be attributed to CVD.^1^ In 2008 and 2009, the two most recent years for which South African data are available, CVD was responsible for 13.7 and 14.0% of total deaths, respectively.^2^-^4^ However, CVD mortality rates are expected to rise in South Africa as unhealthy lifestyle trends associated with urbanisation spread to the countryside, and the population of people surviving life-threatening infections continues to grow.^5^,^6^

Well-known risk factors for CVD include age, gender, dyslipidaemia, tobacco smoking, high blood pressure and diabetes mellitus (DM). Other lifestyle behaviours such as excessive alcohol consumption, sedentary lifestyle and poor diet with resultant obesity further contribute to CVD risk.^7^,^8^ The WHO 2008 estimates indicated that the prevalence of obesity, tobacco smoking and physical inactivity in South Africa were 31.3 (≥ 20 years old), 14 and 51.1%, respectively.^9^ Furthermore, in 2010 the prevalence of DM was 4.5% for individuals ≥ 15 years old,^10^,^11^ and the WHO estimated the rate of high blood pressure at 42.2% in 2008.^9^ As the prevalence of these risk factors rise in South Africa,^5^ so will the rate of CVD.

The main effect of statins is to lower LDL-C levels and they are used extensively in both primary and secondary prevention of CVD.^12^-^14^ Importantly, several large clinical trials have indicated that for every 1-mmol/l reduction in LDL-C levels there is a 23% reduction in CVD risk.^15^-^18^ In a further meta-analysis of studies comparing high and low statin doses, more intensive lowering of LDL-C (0.51 mmol/l additional reduction) in the high-dose statin arm was associated with a further 15% reduction in CVD risk.^19^ In the most recently published statin cardiovascular outcomes trial (JUPITER study: men and women free of overt cardiovascular disease over the ages of 50 and 60 years, respectively; baseline LDL-C < 3.37 mmol/l and high-sensitivity C-reactive protein of 2 mg/l or more; randomised to rosuvastatin 20 mg/day or placebo), statin treatment was associated with a 39% reduction in primary endpoints (myocardial infarction, stroke, admission to hospital for unstable angina, arterial revascularisation or CV death) in patients with at least one risk factor for DM.^20^

The results of these and other studies have resulted in treatment guidelines recommending progressively lower LDL-C targets.^21^-^23^ However, studies from all over the world have demonstrated that many patients on lipid-lowering therapy do not reach their recommended lipid targets.^24^-^26^ The South African Heart Association (SA Heart) together with the Lipid and Atherosclerosis Society of Southern Africa (LASSA) therefore recently emphasised that intensive management of dyslipidaemia could significantly reduce the South African CVD health burden.^21^

The DYSlipidaemia International Study (DYSIS) is a crosssectional, observational study that has examined the efficacy of lipid-lowering therapies in patients from various regions of the world, including Canada and Europe (11 countries), in order to better characterise predictive factors for dyslipidaemia and CVD.^24^,^25^ Here, as part of DYSIS, we have analysed residual dyslipidaemia in statin-treated South African patients.

## Methods

As part of DYSIS, this epidemiological, observational, cross-sectional study was conducted in South Africa between 1 November and 9 December 2011. Data for the study were collected in the South African private healthcare sector by 16 physicians; 50% were primary-care physicians and 50% were specialised office-based physicians (e.g. cardiologists).

Prior to study initiation, the relevant local ethical review committees approved the study protocol and all patients gave written informed consent before enrolling in the study. Key eligibility criteria were: (1) age of at least 45 years, (2) receiving stable statin therapy for at least 3 months, and (3) fasting for at least 12 hours at the time of visit while on statin therapy. Participating physicians were instructed to include all eligible and consenting patients consecutively.

Patient demographic, lifestyle and clinical characteristics were documented. Lipid levels (total cholesterol, LDL-C, HDL-C and triglycerides) were measured using the CardioChek® device (http://www.cardiocheck.com) at the time of patient enrollment to reliably collect lipid measurements uniformly at all sites. The LDL-C test strip provided measures LDL-C directly across a range of 1.29–5.18 mmol/l in about two minutes.

Additionally, the lipid-lowering regimen at the time of the most recent blood sample was recorded for each patient (in particular, statin type and daily dose) as well as any information regarding other lipid-modifying therapies. The potency of different types of statins was normalised using a calculation that allows benchmarking against six different simvastatin dose levels (5, 10, 20, 40, 80 and 160 mg/day), with potency scores ranging from 1 (5 mg/day simvastatin) to 6 (160 mg/day simvastatin).^23^,^27^

The 2011 ESC guidelines were used to classify CV risk, LDL-C level treatment goals, and sub-optimal HDL-C and triglyceride levels.^21^,^28^ Variables independently associated with dyslipidaemia were evaluated with logistic regression modelling using the following variables: age (≥ 70 years), female gender, family history of premature coronary heart disease (CHD), current tobacco smoker, sedentary lifestyle, alcohol consumption (> 2 units/week), body mass index (BMI) ≥ 30 kg/m^2^, large waist circumference (> 102 cm in men, > 88 cm in women^29^), hypertension, DM, coronary heart disease, cerebrovascular disease, heart failure, peripheral artery disease, systolic/diastolic blood pressure ≥ 140/90 mmHg, simvastatin equivalent dose of either 20 to 40 versus 10 mg/day, or > 40 mg versus 10 mg/day, ezetimibe use, and physician’s specialty (cardiologist, endocrinologist, diabetologist, internal medicine or other).

## Statistical analysis

To estimate the sample size needed for South Africa we assumed a prevalence of residual lipid abnormalities between 20 and 60% in patients fulfilling the entry criteria for this study and a design effect of 20% (variance inflation due to cluster sampling design). We calculated that, within this range, a sample size of 1 000 would be sufficient to estimate the prevalence of residual dyslipidaemia with a given precision of ± 3.4% (range of 95% confidence interval: 6.8%). Furthermore we determined that this size guaranteed enough information for estimating the prevalence in smaller subgroups (representing one-quarter or more of the population) with a precision of ± 6.8% (95% CI: 13.6%).

Following data collection, patient information was entered into a central web-based database housed and managed at the Institut für Herzinfarktforschung, Ludwigshafen, Germany. Real-time quality control (internal logic checks) occurred during web-based data entry. Continuous variables are presented as means with standard deviations or medians with 25th and 75th percentiles [interquartile range (IQR)] as indicated, and categorical variables are reported as absolute numbers and percentages.

Kernel density estimation was used to analyse the distribution of total cholesterol, LDL-C, HDL-C and triglyceride levels. The value of a kernel density and its slope at the lipid value equal to the ESC goal provides a crude indicator of the change in the proportions of patients meeting the goal from a small improvement or deterioration in lipid level starting from the ESC goal. This approach thus provides a sensitivity analysis for either changes in the ESC goals or changes in lipid levels for people whose levels are near the goals.

Multiple logistic regression analyses with backward selection (α = 0.05) were used to identify variables independently associated with LDL-C, HDL-C and triglyceride irregularities. Two-tailed statistical comparisons were used (*p* < 0.05 was significant) and patients lacking the appropriate lipid parameters were not included within the analyses. All analyses were performed using SAS v 9.1 (SAS Institute Inc, USA).

## Results

Patient characteristics, risk categories and lipid parameters are presented in Table 1. The study enrolled 1 029 patients (429 men, 600 women). The mean age of patients was 65.4 years, and 58.3% were female. The study population was of mixed ethnic (multi-racial) origin, including Caucasians (56.6%), blacks (22.0%), Asians (9.5%) and patients of mixed ancestry (12.0%).

**Table 1:** Patient Characteristics, Risk Categories And Lipid Parameters In Different Ethnic Groups

	*All patients (n = 1 029)*	*Caucasian (n = 582; 56.6%)*	*Black (n = 226; 22.0%)*	*Asian (n = 99; 9.6%)*	*Mixed ancestry (n = 122; 11.9%)*
Age (years) (mean ± SD)	65.4 ± 10.8	69.0 ± 11.0	60.0 ± 8.9	61.8 ± 9.0	60.9 ± 7.4
Family history of premature CHD (%)	26.7	34.0	1.8	44.4	23.0
Current smokers (%)	10.7	11.2	5.3	11.1	18.0
Hypertension (%)	76.8	69.8	93.3	64.6	89.3
Systolic BP (mmHg) (mean ± SD)	134.4 ± 20.0	134.9 ± 20.4	135.2 ± 19.4	129.0 ± 17.1	134.9 ± 20.7
Diastolic BP (mmHg) (mean ± SD)	79.7 ± 11.0	79.6 ± 11.1	79.6 ± 11.5	78.3 ± 9.6	81.2 ± 10.5
Waist circumference (cm) (mean ± SD)	100.7 ± 15.1	99.5 ± 16.5	105.0 ± 13.4	96.1 ± 9.6	101.8 ± 12.5
BMI (kg/m^2^) (mean ± SD)	29.6 ± 6.4	28.6 ± 6.4	32.8 ± 6.5	27.0 ± 4.5	30.4 ± 5.6
BMI > 30 kg/m^2^ (%)	42.2	36.8	61.9	22.2	47.5
CVD (%)	36.2	41.1	9.7	51.5	49.2
Diabetes mellitus (%)	40.4	25.6	71.2	44.4	50.8
Metabolic syndrome (IDF) (%)	67.2	59.8	83.2	59.8	78.7
ESC risk level (2011)*					
Very high-risk patient (%)	73.5	69.9	77.9	73.7	82.0
High-risk patient (%)	8.9	11.2	4.0	11.1	5.7
Moderate-risk patient (%)	13.5	15.6	11.5	9.1	10.7
Low-risk patient (%)	4.1	3.3	6.6	6.1	1.6
South African guidelines					
Very high-risk patient (%)	68.6	61.2	77.9	73.5	82.8
High-risk patient (%)	9.2	11.7	6.2	8.2	3.3
Moderate-risk patient (%)	21.6	26.6	15.9	15.3	13.9
Low-risk patient (%)	0.6	0.5	0.0	3.1	0.0
Lipids (mmol/l) (mean ± SD)					
LDL-C	2.3 ± 1.1	2.2 ± 1.0	2.1 ± 1.0	2.6 ± 1.2	2.7 ± 1.1
HDL-C	1.3 ± 0.4	1.3 ± 0.4	1.4 ± 0.4	1.3 ± 0.4	1.3 ± 0.5
Total cholesterol	4.4 ± 1.3	4.4 ± 1.2	4.4 ± 1.4	4.7 ± 1.6	4.7 ± 1.3
Triglycerides [median (IQR)]	1.6 (1.1–2.3)	1.5 (1.1–2.2)	1.7 (1.2–2.4)	1.7 (1.2–2.7)	1.5 (1.1–2.4)
Blood glucose					
FBG (mmol/l) [median (IQR)]	4.9 (4.3–6.4)	4.6 (4.2–5.4)	6.2 (4.7–9.0)	5.3 (4.2–7.0)	5.6 (4.7–7.2)
HbA_1c_ (%) diabetics [median (IQR)]	7.4 (6.6–8.8)	7.1 (6.0–8.0)	8.2 (6.8–9.9)	7.8 (7.0–8.7)	7.4 (7.0–8.8)

CHD, coronary heart disease; BP, blood pressure; BMI, body mass index; CVD, cardiovascular disease; DM, diabetes mellitus; IDF, International Diabetes Federation.

Patient characteristics and cardiovascular risk profile differed by ethnic group. A family history of premature CVD was reported by 34% of Caucasian patients while the diabetes prevalence of 25.6% was the lowest of all the ethnic groups studied. Hypertension was found in 69.8% and CVD in 41.1% of Caucasian patients. Black patients were least likely (1.8%) to report a family history of premature CHD and had the lowest (5.3%) smoking rates. However, hypertension was almost universal (93.3%) and diabetes and obesity were highly prevalent at 71.2 and 61.9%, respectively. Despite the high prevalence of hypertension and obesity, only 9.7% of black patients had clinically overt CVD.

Asian patients had the highest rates of CVD (51.5%) among all ethnic groups studied and also the highest reported rate of a family history of premature CVD (44%). Diabetes was highly prevalent at 44.4% while the hypertension prevalence of 64.6% was similar to that observed in Caucasian patients. Mixed-ancestry patients had the highest smoking rates (18%) while the diabetes and hypertension prevalences were 50.8 and 89.3%, respectively. CVD was documented in 49.2% of mixed-ancestry patients.

CVD was almost twice as common in men (49.9%) than women (26.3%). DM was more common in men than women (45.7 vs 36.7%), while obesity was more frequent in women (46.8 compared with 35.7%). Additionally, using the 2011 ESC criteria, 73.5% of patients (83.9% men and 66.0% of women) were classified as very high risk for CV complications [defined as having CVD, DM and/or an ESC systematic coronary risk evaluation (SCORE) risk of ≥ 10% on chronic statin therapy].

## Lipid-modifying regimens and statin potency

Prior to enrollment in DYSIS, patients had been treated with various lipid-lowering therapies. The most commonly prescribed statin was simvastatin (64.6%), followed by atorvastatin (22.2%), rosuvastatin (10.9%), pravastatin (1.6%), fluvastatin (0.6%) and lovastatin (0.2%). Other lipid-lowering agents were used by only 2% of patients, including ezetimibe (1.2%), fibrates (0.9%) and bile acid sequestrants (0.2%).

**Fig. 1.**
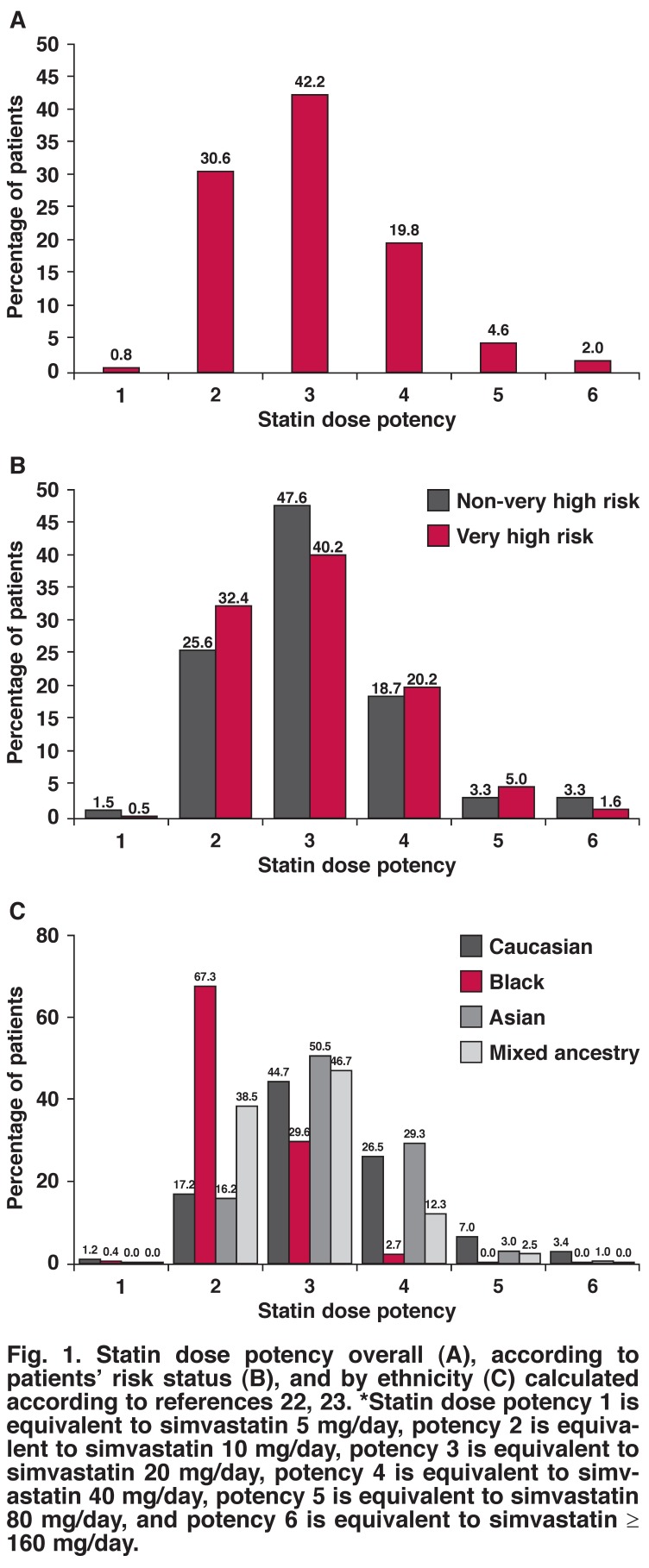
Statin dose potency overall (A), according to patients’ risk status (B), and by ethnicity (C) calculated according to references 22, 23. *Statin dose potency 1 is equivalent to simvastatin 5 mg/day, potency 2 is equivalent to simvastatin 10 mg/day, potency 3 is equivalent to simvastatin 20 mg/day, potency 4 is equivalent to simvastatin 40 mg/day, potency 5 is equivalent to simvastatin 80 mg/day, and potency 6 is equivalent to simvastatin ≥ 160 mg/day.

The most frequently used statin dose potency was 3 (equivalent to 20 mg simvastatin per day) for both very high-risk patients (40.2%) and non-very high-risk patients (47.6%), while the second most-frequent dose potency was 2 (equivalent to 10 mg simvastatin per day) in 32.4 and 25.6% of very high-risk patients and non-very high-risk patients, respectively (Fig. 1). While a statin dose potency of 3 was most frequently used in Caucasian, Asian and mixed-ethnicity patients, a dose potency of 2 was most common in black patients.

## Lipid abnormalities

Data on the frequency of lipid abnormalities, including sub-analyses by CVD risk level, are provided in Tables 2 and 3. Among all patients (*n* = 1 029), 50.3% had LDL-C levels not at goal. We defined ‘not at LDL-C goal’ as LDL-C ≥ 1.8 mmol/l and LDL-C reduction of < 50% for patients with CVD, DM and/or a SCORE risk of ≥ 10% (very high risk), and as ≥ 2.5 mmol/l and ≥ 3 mmol/l for patients with a SCORE risk of 5 to 9% (high risk) and 1 to 4% (moderate risk), respectively. Elevated TG levels (defined as > 1.7 mmol/l) were seen in 45.3% of patients, and 33.7% had low HDL-C levels (defined as < 1.0 mmol/l for men and < 1.2 mmol/l for women).

**Table 2:** Lipid Abnormalities According To ESC Guidelines (2011) On Risk Stratification

	*All patients (n = 1 029)*	Very high risk* (n = 756)	*High risk (n = 92)*	*Moderate risk (n = 139)*	*Low risk (n = 42)*
LDL-C not at target (%)^†+^	50.3	60.1	33.3	24.1	
Low HDL-C [< 1.0 (men)/1.2 (women) mmol/l) (%)^‡^	33.7	36.0	25.0	26.3	34.1
Elevated TG (> 1.7 mmol/l) (%)^§^	45.3	45.8	46.7	40.1	50.0

*Very high risk = CVD, diabetes, and/or SCORE risk ≥ 10% (chronic kidney disease was not documented in DYSIS) ^†^LDL-C ≥ 3.0 mmol/l in patients with SCORE risk 1–4%, LDL-C ≥ 2.5 mmol/l in patients with SCORE risk 5–9%, LDL-C ≥ 1.8 mmol/l in patients with CVD, DM, and/or SCORE risk ≥ 10%; LDL-C ≥ 1.8 mmol/l ^+^Data on 987 patients were available ^‡^Data on 1 025 patients were available ^§^Data on 1 027 patients were available In the ESC 2011 guidelines, no LDL-C goal was specified for the low-risk group.

**Table 3:** Lipid Abnormalities According To ESC Guidelines (2011) In Very High-Risk Patients

	*CVD + DM (n = 131)*	*CVD (w/o DM) (n = 241)*	*DM (w/o CVD) (n = 285)*	*SCORE ≥ 10% (n = 99)*
LDL-C ≥ 1.8 mmol/l and LDL-C reduction < 50% (%)*	57.9	68.0	53.8	61.9
Low HDL-C [< 1.0 (men)/1.2 (women) mmol/l] (%)^†^	39.7	33.8	37.9	31.3
Elevated TG (> 1.7 mmol/l) (%)^‡^	54.2	38.2	51.9	35.4

*Data on 722 of a total of 756 high-risk patients were available ^†^Data on 755 patients were available ^‡^Data on 756 patients were available.

The most prevalent lipid disorder (either alone or in combination) in very high-risk patients was above-target LDL-C levels (60.1%), followed by elevated TG levels (45.8%), and low HDL-C levels (36.0%). By contrast, in both high- and moderate-risk patients, elevated TG levels were observed more frequently (46.7 and 40.1%, respectively in the two risk groups) than above-target LDL-C levels (33.3 and 24.1%, respectively) and low HDL-C levels (25.0 and 26.3% for both risk groups, respectively).

We next performed a sub-analysis of lipid abnormalities for only very high-risk patients (756 of all patients, Table 3), which we stratified as indicated. Of those with CVD and DM, 57.9% displayed off-target LDL-C levels (≥ 1.8 mmol/l and a decrease in LDL-C levels of < 50%), 39.7% showed low HDL-C levels, and 54.2% had elevated TG levels. In comparison, patients in the CVD without DM group showed a higher rate of LDL-C not at target (68.0%), decreased rates of low HDL-C (33.8%), and elevated TG levels (38.2%). Interestingly, the ESC SCORE group with risk of ≥ 10% showed a lower proportion of patients with low HDL-C and elevated TG levels. Overall, we found that LDL-C not at goal was the most common lipid abnormality observed in each of the four sub-sets.

**Fig. 2.**
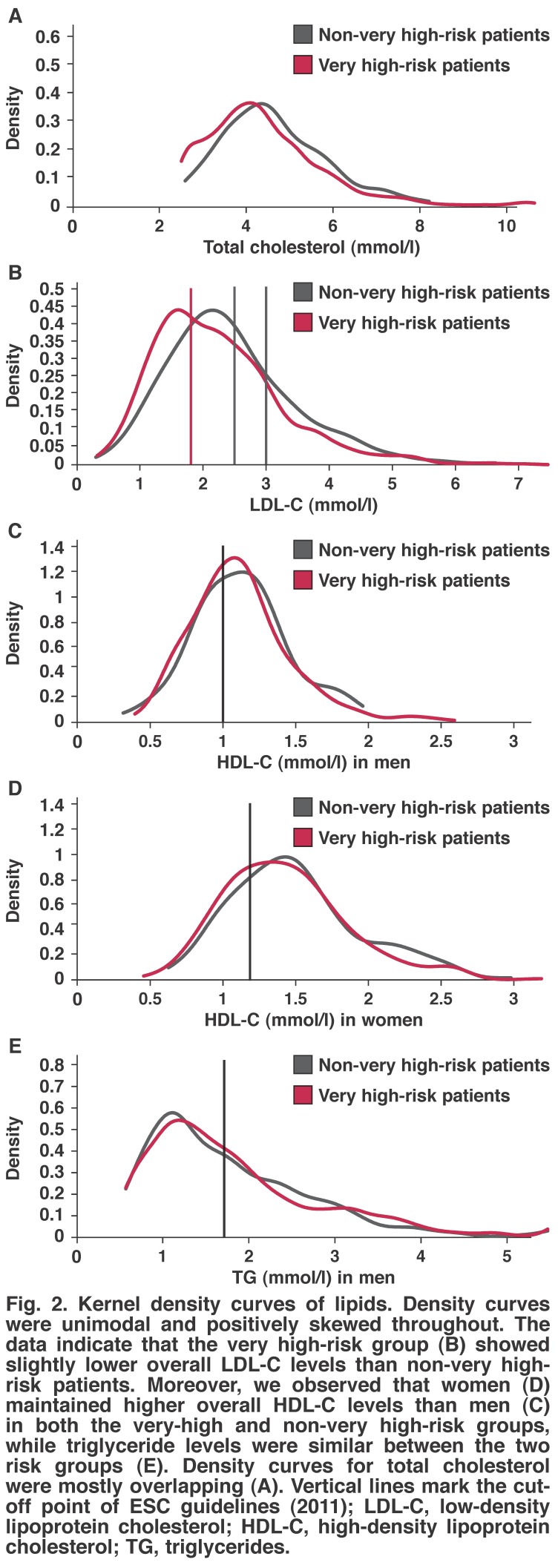
Kernel density curves of lipids. Density curves were unimodal and positively skewed throughout. The data indicate that the very high-risk group (B) showed slightly lower overall LDL-C levels than non-very high-risk patients. Moreover, we observed that women (D) maintained higher overall HDL-C levels than men (C) in both the very-high and non-very high-risk groups, while triglyceride levels were similar between the two risk groups (E). Density curves for total cholesterol were mostly overlapping (A). Vertical lines mark the cut-off point of ESC guidelines (2011); LDL-C, low-density lipoprotein cholesterol; HDL-C, high-density lipoprotein cholesterol; TG, triglycerides.

Additionally, we analysed patient lipid abnormalities using kernel density curves for the empirical distributions of very high-risk and non-very high-risk patient groups with regard to total cholesterol, LDL-C, HDL-C (separately for men and women), and TG levels (ESC guidelines indicated as superimposed vertical lines) (Fig. 2). Overall, we found that the density curves were unimodal and positively skewed, and the data indicated that the very high-risk group showed slightly lower overall LDL-C levels than non-very high-risk patients. Moreover, we observed that women maintained higher overall HDL-C levels than men in both the very high and non-very high-risk groups, while TG levels were similar between the two risk groups.

## Distributions of lipid abnormalities

**Fig. 3.**
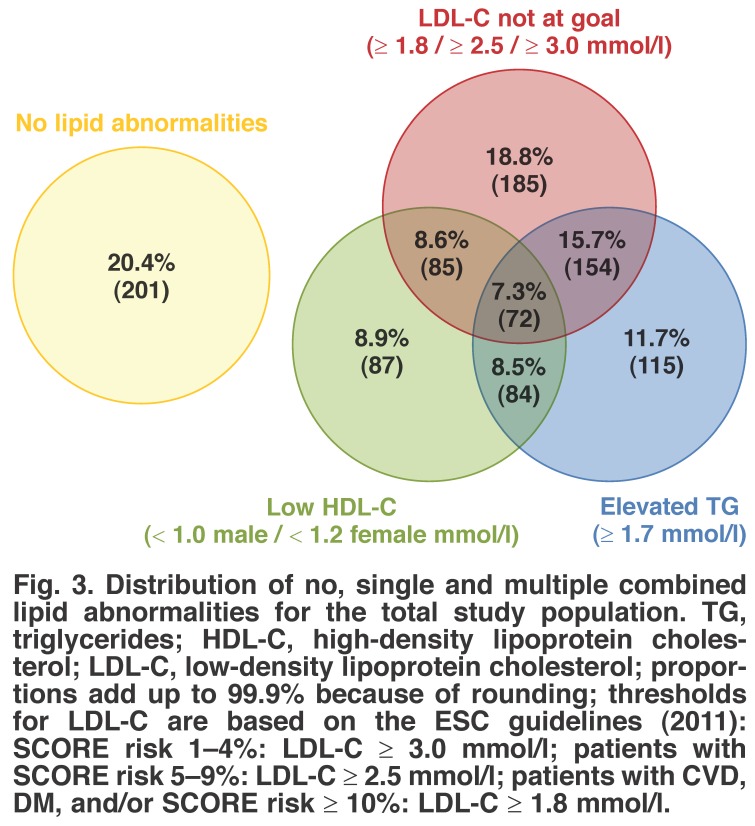
Distribution of no, single and multiple combined lipid abnormalities for the total study population. TG, triglycerides; HDL-C, high-density lipoprotein cholesterol; LDL-C, low-density lipoprotein cholesterol; proportions add up to 99.9% because of rounding; thresholds for LDL-C are based on the ESC guidelines (2011): SCORE risk 1–4%: LDL-C ≥ 3.0 mmol/l; patients with SCORE risk 5–9%: LDL-C ≥ 2.5 mmol/l; patients with CVD, DM, and/or SCORE risk ≥ 10%: LDL-C ≥ 1.8 mmol/l.

**Fig. 4.**
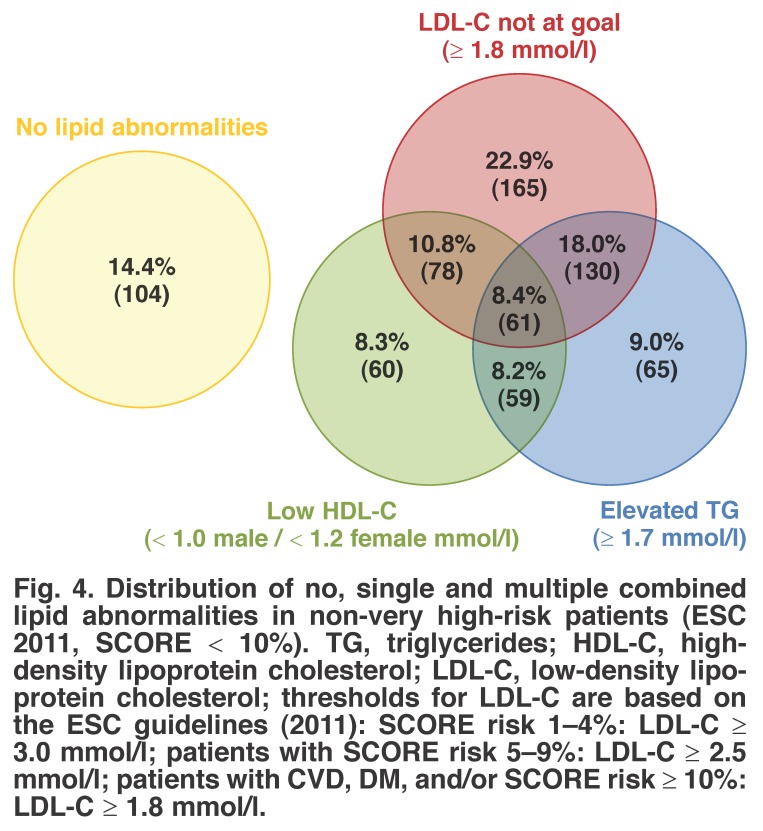
Distribution of no, single and multiple combined lipid abnormalities in non-very high-risk patients (ESC 2011, SCORE < 10%). TG, triglycerides; HDL-C, highdensity lipoprotein cholesterol; LDL-C, low-density lipoprotein cholesterol; thresholds for LDL-C are based on the ESC guidelines (2011): SCORE risk 1–4%: LDL-C ≥ 3.0 mmol/l; patients with SCORE risk 5–9%: LDL-C ≥ 2.5 mmol/l; patients with CVD, DM, and/or SCORE risk ≥ 10%: LDL-C ≥ 1.8 mmol/l.

**Fig. 5.**
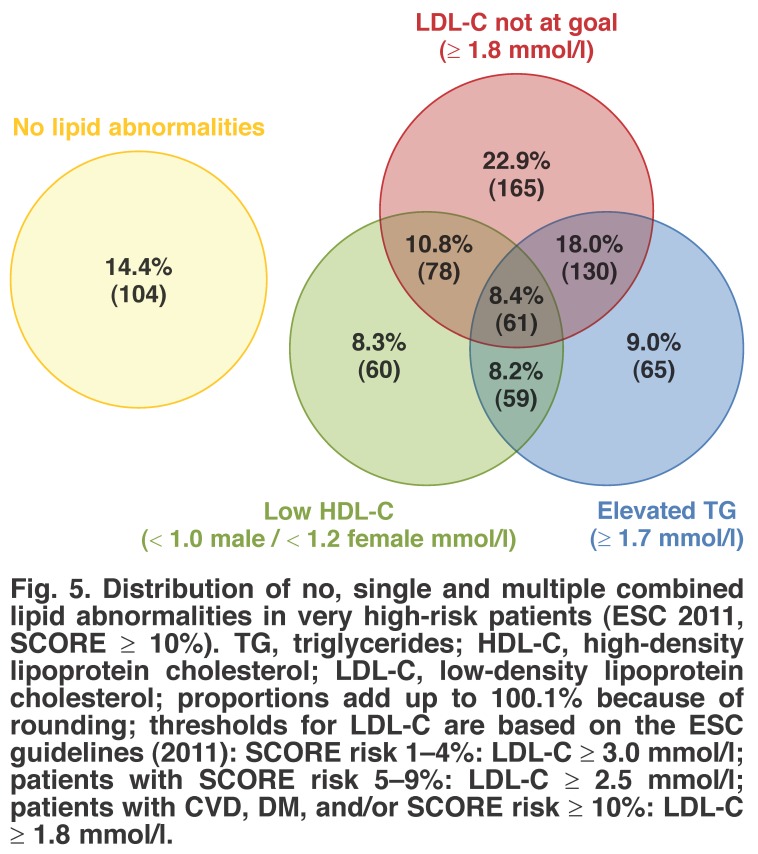
Distribution of no, single and multiple combined lipid abnormalities in very high-risk patients (ESC 2011, SCORE ≥ 10%). TG, triglycerides; HDL-C, high-density lipoprotein cholesterol; LDL-C, low-density lipoprotein cholesterol; proportions add up to 100.1% because of rounding; thresholds for LDL-C are based on the ESC guidelines (2011): SCORE risk 1–4%: LDL-C ≥ 3.0 mmol/l; patients with SCORE risk 5–9%: LDL-C ≥ 2.5 mmol/l; patients with CVD, DM, and/or SCORE risk ≥ 10%: LDL-C ≥ 1.8 mmol/l.

Distributions of single and multiple combined lipid abnormalities for our study are shown in Figs 3, 4, 5. Here, we present the joint distribution of lipid abnormalities for the entire sample and then for sub-samples of very high-risk and non-very high-risk patients. Additionally, joint distributions that either include or exclude patients with no lipid abnormalities are provided for each patient group.

Fig. 3 shows that in 39.4% of patients with a total lipid profile, there was only one single-lipid abnormality, 32.8% had two abnormalities, and the remaining 7.3% had abnormalities in all three assessed components of the lipid profile. Among statin-treated patients, the most common abnormality was high LDL-C levels (18.8% of all cases), accounting for 47.7% of all single-lipid abnormalities. Among the 983 patients, 20.4% had no lipid abnormalities.

Figs 4 and 5 present the joint distribution for non-very high-risk and very high-risk patients, respectively, and indicate different patterns of prevalence for these sub-groups. For the 261 non-very high-risk patients with at least one abnormality depicted in Fig. 4, 37.2% had only one lipid abnormality, 21.5% had two lipid abnormalities and the remaining 4.2% had all three lipid abnormalities.

By contrast, for the 826 very high-risk patients depicted in Fig. 5, the majority, 45.4%, had two or more lipid abnormalities (40.2% had one, 37.0% had two, and the remaining 8.4% had all three). For non-very high-risk patients, elevated triglycerides were the largest single abnormality present, appearing in 42.2% of all non-very high-risk patients. By contrast, among very high-risk patients, high LDL-C level was the most frequent abnormality, at 60.1% of all very high-risk patients.

## Variables independently associated with dyslipidaemia

Multivariate logistic regression analyses indicated that among the 19 risk factors incorporated into the model, mixed ancestry, along with history of hypertension, DM and cerebrovascular disease were among the risk factors strongly, positively and independently associated with LDL-C levels not being at goal. Having low HDL-C levels was negatively associated with female gender and increased alcohol consumption, but positively associated with being treated by a specialist, increased waist circumference, and presence of DM. Having elevated triglyceride levels was negatively associated with age above 70 years, but positively associated with female gender, obesity, history of DM and peripheral artery disease. The three variables independently associated with having all three lipid abnormalities were Asian and mixed-ancestry ethnicity versus Caucasian ethnicity, and obesity, all of which were positively associated with not reaching goal (Table 4).

**Table 4:** Factors Independently Associated With LDL-C, HDL-C And TG Abnormalities: Results From Multiple Regression Analyses (Or, 95% CI)

	LDL-C not at target*† (≥ 1.8/2.5/3.0 mmol/l)	Low HDL-C* [< 1.0 (m)/1.2 (w) mmol/l]	Elevated TG* (> 1.7 mmol/l)	LDL-C not at target, low HDL-C, elevated TG*
Age ≥ 70 years	ns	ns	0.57 (0.43–0.77)	ns
Female	ns	0.43 (0.32–0.58)	1.33 (1.02–1.74)	ns
Asian vs Caucasian	ns	ns	ns	2.48 (1.19–5.16)
Black vs Caucasian	ns	ns	ns	ns
Mixed ancestry vs Caucasian	2.12 (1.36–3.32)	ns	ns	2.78 (1.50–5.19)
Alcohol consumption > 2 units/week	ns	0.50 (0.31–0.79)	ns	ns
BMI ≥ 30 kg/m^2^ (obesity)	ns	ns	1.74 (1.33–2.29)	2.11 (1.27–3.50)
WC > 102 (m)/> 88 cm (w)	ns	1.71 (1.26–2.32)	ns	ns
Hypertension	1.55 (1.12–2.13)	ns	ns	ns
Diabetes mellitus	1.36 (1.01–1.82)	1.58 (1.17–2.15)	1.49 (1.12–1.98)	ns
Cerebrovascular disease	1.89 (1.39–2.57)	ns	ns	ns
Peripheral artery disease	ns	ns	2.35 (1.09–5.07)	ns
Specialist (Card/Endo/Dia/Int/Oth)	ns	2.01 (1.46–2.76)	ns	ns

*Models contained the following variables: age, gender, ethnicity, 1st-grade family history of premature CVD, current smoker, sedentary lifestyle, alcohol consumption > 2 units/week, BMI ≥ 30 kg/m^2^ (obesity), waist circumference > 102 cm in men/> 88 cm in women, hypertension, diabetes mellitus, coronary heart disease, cerebrovascular disease, heart failure, peripheral artery disease, RR ≥ 140/90 mmHg (systolic/diastolic), 20–40 vs 10 mg/day simvastatin equivalent, ≥ 80 vs 10 mg/day simvastatin equivalent, ezetimibe. Backward selection (alpha = 0.05) was done. ^†^Patients with SCORE risk 1–4%: LDL-C ≥ 3.0 mmol/l; patients with SCORE risk 5–9%: LDL-C ≥ 2.5 mmol/l; patients with CVD, DM, and/or SCORE risk ≥ 10%: LDL-C ≥ 1.8 mmol/l Card = cardiologist, Endo = endocrinologist, Dia = diabetologist, Int = internist, Oth = other speciality, ns = not significant (p > 0.05), OR = odds ratio, CI = confidence interval.

## Discussion

In the DYSIS South Africa study we observed marked ethnic differences in cardiovascular risk profiles and the primary indication for statin therapy. While about half of Asian and mixed-ancestry patients had clinically overt CVD, the rate in black patients was less than 10%. The major indication for statin therapy in black patients was diabetes, which was present in 71.2% of patients. A family history of premature CVD was very uncommon (1.8%) in black patients.

These data are reflective of the epidemiological transition, which the South African black population is currently undergoing,^6^ with increasing urbanisation and transition to a Westernised lifestyle. Hypertension, obesity and diabetes are highly prevalent in black patients while CVD, which results from prolonged exposure to cardiovascular risk factors, is still relatively uncommon. With further progression of the epidemiological transition, CVD rates in black patients are likely to rise and may well match or exceed those observed in the other ethnic groups if cardiovascular risk factors are not addressed intensively, both on a population and an individual level.

The DYSIS South Africa study identified a group of patients at high cardiovascular risk, with 73.5% of statin-treated patients assessed to be at very high risk for CVD. Within this very high-risk group, despite statin therapy, 85.6% had at least one lipid abnormality, of which a majority had two or more lipid abnormalities. The most common lipid abnormality was high LDL-C levels, which was diagnosed in 60.1% of all very high-risk patients.

Moreover, for all patients in the study, 50.5% had LDL-C levels not at goal, which is comparable with the findings from the recently published CEPHEUS-SA study and the Canadian/European cohort of the DYSIS study, and below the levels found in the Middle Eastern cohort (62%).^24^,^26^,^30^ Not surprisingly, the metabolic syndrome was present in 67.2% of the sample, since its components also contribute to elevated CVD risk.

Statistically significant factors associated with high LDL-C levels included ethnicity, hypertension, DM, and the presence of coronary and cerebrovascular heart disease. Factors associated with low HDL-C levels were a high waist circumference, DM and being treated by a specialist. Elevated TG was associated with female gender, obesity, DM and peripheral artery disease. However, the only statistically significant factors independently associated with the presence of all three lipid abnormalities were obesity and Asian as well as mixed-ancestry ethnicity.

Based on the current data, it is unclear whether the findings with regard to ethnicity are biologically or sociologically determined. Even though this study was conducted exclusively in the private healthcare sector in South Africa, Asian or mixed-ancestry ethnicity most likely still correlates partially with social deprivation, which has been shown to be a risk factor for cardiovascular disease. Social deprivation may also affect access to medical care, with less access to specialist care and a bias towards less aggressive treatment. Studies from other countries have shown that ethnic minorities or immigrants often receive less aggressive cardiovascular care,^31^ as also observed in this study, with black patients receiving lower-dose potency of statins, despite the majority of patients being at high risk.

Socio-economic status has also been associated with statin adherence,^32^ as has ethnicity.^33^ In the South African context, lower socio-economic status would, for instance, often correlate with membership of a medical scheme option that restricts lipid-lowering treatment to less-potent (and less-costly) options. Lower income may also influence the willingness and ability to pay ‘co-payments’ that are often required to access more potent lipid-lowering therapy. However, factors such as provider bias, access to treatment and differential adherence do not completely explain the observed ethnic differences, as black patients generally still experience the highest level of socio-economic deprivation as a legacy of South Africa’s past history.

Lesser goal attainment may also in part be due to differences in baseline lipids. In the Heart of Soweto study, there were significant differences in untreated lipid profiles by ethnicity in patients presenting for cardiovascular care^34^ at a tertiary referral centre. The odds ratio (compared to black patients) for elevated LDL-C levels in Asian and mixed-ancestry patients was 4.66 and 2.44, respectively. Indian and mixed-ancestry patients also had higher median TG levels (1.8 and 1.4 mmol/l, respectively) than black patients (1.1 mmol/l).

In addition to identifying factors that are associated with dyslipidaemia in statin-treated patients, DYSIS in South Africa (along with previous DYSIS studies) also highlights the deficiencies of lipid-lowering therapy in clinical practice. Other researchers analysing the efficacy of lipid-lowering therapies have supported this conclusion,^35^,^36^ including another recent study analysing statin-treated South African patients.^26^ Together, these findings suggest that there is a need to improve upon existing treatment strategies (e.g. combination of current therapies for optimal patient efficacy, utilisation of more-potent statins, improving adherence) while also developing novel therapeutic approaches.

Combination therapies were evaluated in the Austrian Cholesterol screening and Treatment (ACT) II study, which evaluated the effect of lipid-lowering therapies in high-risk, statin-treated patients with elevated LDL-C levels. Interestingly, combination therapy consisting of simvastatin and ezetimibe (used for 73% of patients in the ACT II study) resulted in 40.3% of patients meeting their LDL-C goals, with a decline in LDL-C levels from a baseline of 31.3% following 12 months of intensified therapy.^37^

High-dose statins are another option to achieve LDL-C targets in high-risk patients.^38^,^39^ Improving adherence is a challenge that physicians face every day, and some strategies that have shown promise include regular phone calls by a practice nurse, regular review by a community pharmacist and providing a medication calendar when patients filled their first prescription.^40^ There is likely no single strategy that will work for all patients but studies show that adherent patients have much better cardiovascular outcomes than non-adherent patients, although some of the improvement may also be ascribed to the correlation between adherence and other healthy behaviours.^41^-^44^

According to a mathematical model of statin use in a population, increasing statin adherence from 50 to 75% at five years would prevent more events than lowering the risk threshold for prescribing statins.^45^ Lastly, novel LDL-C-lowering therapies may be necessary for patients with very high baseline LDL-C levels, such as is seen in familial hypercholesterolaemia, and when patients are unable to tolerate adequate doses of potent statins.^46^

In South Africa, the modal statin dose potency prescribed to patients was 3, which was prescribed to 42.2% of the individuals. Interestingly, although the very high-risk patients had a disproportionately high share of the statin prescriptions with a potency of 4 and 5, they also had a disproportionately high amount of prescriptions with a potency of 2, and a disproportionately low share of the prescriptions with a potency of 6. In addition, although combination therapies may have the potential to benefit some patients,^37^ we found that the use of combination treatment with lipid-lowering therapies was rare in South Africa. Only seven patients were co-prescribed statins and ezetimibe in this study.

DYSIS-South Africa had several limitations, including its cross-sectional design, which did not permit follow up to assess the effects of statins over time in either reducing CVD risk factors or their ultimate effects in reducing CVD. In addition, the cross-sectional nature of the study precludes us from drawing conclusions of temporality based on observed associations. The study was also only conducted in the private sector and does not therefore provide any information on the care provided in the public sector, which accounts for about 80% of patients in South Africa. As this study was conducted in the private sector, the ethnic make-up of the DYSIS study cohort is not representative of the South African population at large.

Furthermore physicians were aware of the study purpose, possibly making the results prone to a selection bias towards patients with better-than-average lipid goal attainment. DYSIS by its design is also unable to provide data on the important public health question on what proportion of patients with an indication for lipid-lowering therapy is actually being treated. Analysing patients that return for follow-up consultation and are still taking statins is not reflective of the entire statin treatment experience, as patients discontinuing early and defaulting on follow up are not captured. However, in spite of these potential limitations, the data obtained during this cross-sectional, observational study of South Africa has furthered our knowledge of CV risk and the factors that contribute to persistent dyslipidaemia in statin-treated patients.

## Conclusions

The DYSIS study for South Africa, like the DYSIS studies in other countries and regions, indicates that large proportions of statin-treated patients have persisting lipid abnormalities, which place them at ongoing risk for CVD. While some observations with regard to co-morbid conditions and demographics associated with lipid goal attainment were expected, observations also demonstrate a decreased likelihood of obtaining lipid goals among two ethnic minority groups, independent of treatment, demographics and other co-morbidities. These findings deserve further attention. As statins remain among the most effective agents for preventing CVD, the findings of this study emphasise the necessity for more aggressive therapy in order to achieve recommended lipid targets, so as to reduce the burden of cardiovascular disease, which is on the increase not only in South Africa but worldwide.
